# Structural Characterization of *Mycobacterium tuberculosis* Encapsulin in Complex with Dye-Decolorizing Peroxide

**DOI:** 10.3390/microorganisms12122465

**Published:** 2024-11-30

**Authors:** Bonnie J. Cuthbert, Xiaorui Chen, Kalistyn Burley, Gaëlle Batot, Heidi Contreras, Shandee Dixon, Celia W. Goulding

**Affiliations:** 1 Department of Molecular Biology and Biochemistry, University of California Irvine, Irvine, CA 92697, USA; 2 Department of Pharmaceutical Sciences, University of California Irvine, Irvine, CA 92697, USA

**Keywords:** *Mycobacterium tuberculosis*/pathogenicity, *Mycobacterium tuberculosis*/enzymology, bacterial proteins, crystallography, X-ray, peroxidase

## Abstract

*Mycobacterium tuberculosis* (Mtb) is the causative agent of tuberculosis, the world’s deadliest infectious disease. Mtb uses a variety of mechanisms to evade the human host’s defenses and survive intracellularly. Mtb’s oxidative stress response enables Mtb to survive within activated macrophages, an environment with reactive oxygen species and low pH. Dye-decolorizing peroxidase (DyP), an enzyme involved in Mtb’s oxidative stress response, is encapsulated in a nanocompartment, encapsulin (Enc), and is important for Mtb’s survival in macrophages. Encs are homologs of viral capsids and encapsulate cargo proteins of diverse function, including those involved in iron storage and stress responses. DyP contains a targeting peptide (TP) at its C-terminus that recognizes and binds to the interior of the Enc nanocompartment. Here, we present the crystal structure of the Mtb-Enc•DyP complex and compare it to cryogenic-electron microscopy (cryo-EM) Mtb-Enc structures. Investigation into the canonical pores formed at symmetrical interfaces reveals that the five-fold pore for the Mtb-Enc crystal structure is strikingly different from that observed in cryo-EM structures. We also observe DyP-TP electron density within the Mtb-Enc shell. Finally, investigation into crystallographic small-molecule binding sites gives insight into potential novel avenues by which substrates could enter Mtb-Enc to react with Mtb-DyP.

## 1. Introduction

*Mycobacterium tuberculosis* (Mtb), the causative agent of tuberculosis (TB), is the deadliest infectious killer of humans. In 2023, TB resulted in an estimated 1.3 million deaths and 10.6 million new infections [1]. While TB is treatable, the currently used drug regimens require 4–5 antibiotics, are long in duration, and have numerous side effects [2]. Due to these issues, treatment noncompliance is common, fueling the emergence of multi-drug and extensively drug-resistant Mtb strains [2]. The discovery of novel anti-TB drug targets is an important step in the development of desperately needed new anti-TB therapeutics [3,4]. Deciphering how Mtb interacts with and evades its host is one approach to revealing novel drug targets.

Mtb encapsulin (Enc) has been implicated in the Mtb oxidative stress response, which is critical for Mtb’s survival in the host [5,6]. Enc nanocompartments have also been implicated in a number of metabolic pathways in other bacteria, including iron mineralization and storage [7,8,9,10,11,12], anaerobic ammonium oxidation [13], and sulfur metabolism [14,15,16], in addition to the oxidative stress response [5,6,17,18]. Enc nanocompartments self-assemble to form a protein shell with icosahedral symmetry. The Enc protein is a homolog of a single subunit viral capsid protein and adopts a HK97 (bacteriophage Hong Kong 97)-like fold [19]. Enc nanocompartments are formed from 60 (Triangulation (T) number = 1 [7,9,11,12,17,18,19,20,21,22,23], 180 (T = 3, [7,10,24,25]), or 240 (T = 4, [8,26]) subunits, resulting in protein shells from 21 to 42 nm in diameter [27]. Each Enc has a dedicated protein cargo encapsulated within the Enc shell, where the cargo protein dictates the function of the complex.

To date, a number of different cargo proteins have been identified, including dye-decolorizing peroxidase (DyP), ferritin-like protein (FLP), iron-mineralizing Enc-associated firmicute (IMEF), hemerythrin and rubrerythrin [8,13,16,19,28]. At the N- or C-terminus of the cargo protein there is a targeting domain or targeting peptide (TP) which is between 10 to 40 residues in length and is required for cargo loading [16,19,27]. Cargo loading is generally believed to rely on efficient coexpression and cotranslation of the cargo and encapsulin proteins, where the TP localizes its cargo to the interior of the Enc shell as the nanocompartment assembles [27].

Enc systems have remarkable promise for biotechnology and for drug design and delivery [29,30]. Several characteristics make Enc uniquely attractive for these purposes: (1) the simplicity of the system and potential for genetic engineering, (2) Enc systems can be utilized to encapsulates highly diverse cargo, and (3) the system self-assembles in both prokaryotic and eukaryotic cells [29,30]. Ultimately, Enc can be engineered to deliver non-native enzymes to specific targets. Thus, furthering our understanding of these systems could empower the design of therapeutics.

In Mtb, the operon encoding Enc (Rv0798c) also encodes for DyP (Rv0799c) [5], with the stop codon for Rv0799c overlapping the start codon for Rv0798c. DyP is a heme-containing enzyme that oxidizes and degrades a wide range of synthetic dyes and, importantly, reduces hydrogen peroxide (H_2_O_2_) to water [5]. It has been shown that Mtb-DyP is a hexamer [6], where the C-terminus of Mtb-DyP contains the TP sequence that targets Mtb-DyP to the interior of the Mtb-Enc 60-mer shell [5].

Mtb-Enc•DyP is critical for resisting oxidative stress at low pH, as a Mtb *enc-dyp* deletion strain (Mtb∆*enc-dyp*) exhibits diminished survival at pH 4.5 in the presence of H_2_O_2_ [6]. Mtb requires the expression of both Enc and DyP for full protection against oxidative stress [6], allowing for Mtb’s survival in human macrophages. Notably, Mtb∆*enc-dyp* also shows increased susceptibility to the antibiotic pyrazinamide (PZA) [6], indicating that Mtb-Enc may be a good combination drug target. Despite the clear biological importance of the Mtb-Enc•DyP complex, the biologically relevant substrate of Mtb-DyP has not yet been identified nor has its point of entry into the nanocompartment nor its mechanism of delivery to Mtb-DyP. However, as discussed above, Enc nanocompartments have vast promise for therapeutics and drug delivery, and thus understanding Mtb-Enc•DyP could empower novel mechanisms for therapeutic delivery to Mtb.

Here, we have solved the crystallographic structure of the Mtb-Enc•DyP complex to 3.15 Å resolution. A comparison of the Mtb-Enc crystal structure (PDB ID 9BKX) with previously determined Mtb-Enc cryogenic electron microscopy (cryo-EM) structures (PDB IDs 7PHM, 7P1T, 8IKA, 8PYS) reveals dramatic differences in the pores formed at and around the five-fold symmetry point. While DyP was cocrystallized with Enc, there is only observable electron density for Mtb-DyP-TP. In addition to Mtb-DyP-TP, we also observed density across the Mtb-Enc shell for glycerol, polyethylene glycol (PEG), and nickel ions; small molecules that were introduced to the complex during purification and crystallization. Notably, the observed symmetric pores with bound solvent molecules that traverse the shell may indicate channels by which Mtb-DyP substrate/product(s) could enter or exit the Mtb-Enc interior.

## 2. Materials and Methods

Mtb-Enc_His_ and Mtb-DyP were coexpressed and copurified in BL21 Gold (DE3) cells (Agilent, Santa Clara, CA, USA) as described previously [5]; however, neither the catalytic activity nor the heme content was determined for the crystallized complex. Purified Mtb-Enc•DyP was concentrated to ~11 mg/mL and screened against 600 sparse matrix crystallization conditions using a Mosquito nanoliter dispensing robot (SPT Labtech, Covina, CA, USA) and hanging-drop vapor diffusion techniques. Initial crystals were grown in Qiagen PEGs Suite condition 24: 0.1 M TRIS-HCl pH 8.5, 25% PEG 2000 MME (Germantown, MD, USA). Crystals were further optimized using the Hampton Research additive screen (Aliso Viejo, CA, USA) and crystallization was aided by 3% Trimethlyamine *N*-oxide (TMN). Diffraction data from a single, diffracting crystal grown in 0.1 M TMN, 0.1 M Tris-HCl pH 8.5, and 20% PEG MME 2000 were collected at Stanford Synchrotron Radiation Laboratory (SSRL) beamline 7-1. No cryoprotectant was used.

Diffraction data were processed in iMosflm 7.2.2 to 3.15 Å resolution (Table 1) [31]. Phases were provided by an Enc structure from *Thermatoga maritima* (PDB ID 3DKT, [19]) and solved by molecular replacement (MR) in Phaser (PHENIX 1.9-1692) [32]. The initial model was improved by PHENIX 1.21-5207 AutoBuild [33]. Reiterative rounds of manual refinement in coot (0.8.9.1-1.10.08) and phenix.refine (PHENIX 1.21-5207) resulted in a final R_work_/R_free_ of 20.2/23.3 [34,35]. Electron density maps were produced in Polder (PHENIX 1.21-5207) (Figure S1) [36].

## 3. Results and Discussion

### 3.1. Crystallographic Structure of Mtb-Enc in Complex with DyP

Despite several available cryo-EM structures of Mtb-Enc alone and in complex with DyP, many questions remain regarding the interactions between the two proteins and the Mtb-Enc•DyP system more generally. To address some of these questions*,* Mtb-Enc was coexpressed and copurified with Mtb-DyP and the Mtb-Enc•DyP complex was crystallized. The crystal structure of the Mtb-Enc•DyP complex was solved by MR to 3.15 Å resolution and refined to a final R_work_/R_free_ of 20.2/23.3 (Table 1).

**Figure 1:**
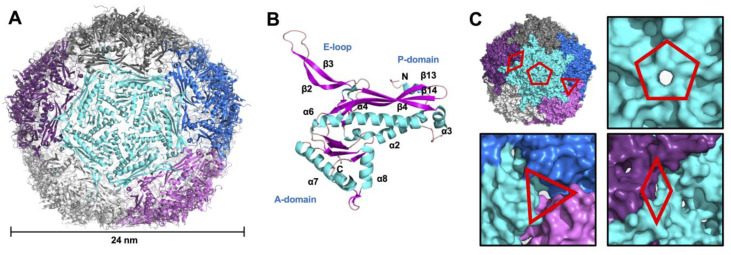
Crystallographic structure of Mtb-Enc•DyP. (**A**) The encapsulin shell generated by crystallographic symmetry is shown in cartoon representation and colored to highlight the five-fold axis. The nanocompartment has a diameter of ~24 nm as measured with Draw Protein Dimensions script [39]. (**B**) Structure of a single subunit of Mtb-Enc in cartoon representation colored by secondary structure: α-helices are in cyan, β-sheets in pink, and loops are wheat-colored. A- and P-domains, and E-loop are labelled, as are N- and C-termini and secondary structure elements. (**C**) Pores formed by two-fold, three-fold, and five-fold symmetry in the Mtb-Enc shell. The molecular surface of the protein is colored as in A. The two-fold, three-fold, and five-fold pores are indicated by diamond, triangle and pentamer symbols, respectively. A closer view of each pore is provided. Figures generated in PyMOL [39].

The resulting structure of Mtb-Enc•DyP (PDB ID 9BKX) contains 20 Mtb-Enc subunits in the asymmetric unit (ASU) (Figure S1A). Application of crystallographic symmetry generated an intact 60-subunit capsule with T1 icosahedral symmetry (Figure 1A). The Mtb-Enc subunit adopts the HK97 phage-like fold consisting of the canonical axial domain (A-domain), peripheral domain (P-domain), and extended loop (E-loop) (Figure 1B). A structural homology search using the Dali server [37] unsurprisingly yielded mycobacteria Enc structures as the top closest structural homologs to Mtb-Enc (Table S1). These included *Mycobacterium hassiacum* (PDB ID 6I9G, 0.4 Å root mean square deviation (rmsd)) and *Mycobacterium smegmatis* (Msm; PDB ID 7BOJ, 0.9 Å rmsd) Enc•DyP structures as the closest matches, both of which have upwards of 80% amino acid sequence similarity to Mtb-Enc, along with the cryo-EM Mtb-Enc structures (PDB IDs 7PHM, 8IKA, and 8PYS, and lastly 7P1T, which was solved in situ and will not be discussed due to unknown cargo, with 0.8–0.9 Å rmsd) (Table S1). The closest non-mycobacteria Mtb-Enc structural homologs were an Enc•DyP complex and apo-Enc from *Klebsiella pneumoniae* (Kpn; PDB ID 8U51 and 8U50, 0.9 Å rmsd) and apo-Enc from *Brevibacterium linens* (PDB ID 7BCV, 1.0 Å rmsd) (Table S1). Notably, the diameters of the mycobacteria and Kpn Enc structures did not vary drastically between DyP-loaded and unloaded structures, in contrast to the biophysical study of *Rhodococcus jostii* Enc [38].

While the compact domain of Mtb-DyP (residues 1-314 based on the AlphaFold Mtb-DyP predicted structure [40]) lacks a corresponding electron density in the interior of the Mtb-Enc shell and cannot be modeled, the electron density for Mtb-DyP-TP was observed (Figure S1C). In the ASU, nine DyP-TPs were modeled (Figure S1). These peptides were bound to the interior of subunits D, G, H, I, P, Q, R, S, and T, and are referred to as d, g, h, i, p, q, r, s, and t, respectively. As is the case for other characterized Enc•DyP complexes [17,18,19], only residues near the conserved TP sequence could be modeled. Overall, the Mtb-DyP-TPs had electron density sufficient to build nine residues on average; however, a range of 6–12 residues were modeled for the Mtb-DyP-TPs (Figure S1D). Natively loaded DyP in the Enc shell generally results in one or two DyP hexamers per icosahedral unit [17,18,19,22]; thus, the nine bound DyP-TP per 20 Enc subunits are not biologically relevant. Instead, we are likely observing an averaging of DyP-TP sites across the entire unit cell. Notably, the *Thermatoga maritima* Enc•FLP crystal structure (PDB ID 3DKT) has a FLP-TP modeled at every Enc subunit in the ASU [19].

In addition to the Mtb-DyP-TPs, the Mtb-Enc•DyP structure displayed excess electron density that we modeled with molecules from the purification and crystallization process: glycerol, nickel (Ni^2+^) ions, and PEG molecules (Figure S1A). Ni^2+^ ions were modeled when waters did not sufficiently fulfill the electron density. When the 20 subunits are superimposed, several locations with a high occurrence of small-molecule binding become apparent (Figure S1B). Notably, one of these locations occurs near the N-terminal region of the modeled DyP-TP (Figure S1E). The proximity of these molecules to bound DyP-TP indicates that these small molecules may be fulfilling poorly resolved electron density for DyP interacting with the interior Enc shell. Notably, a few other small-molecule “clustering” locations extend into and sometimes traverse the Enc shell (Figure S1A,B) and could represent solvent or ligand channels in Mtb-Enc.

### 3.2. Variability of the Five-Fold Pore Across Species

**Figure 2:**
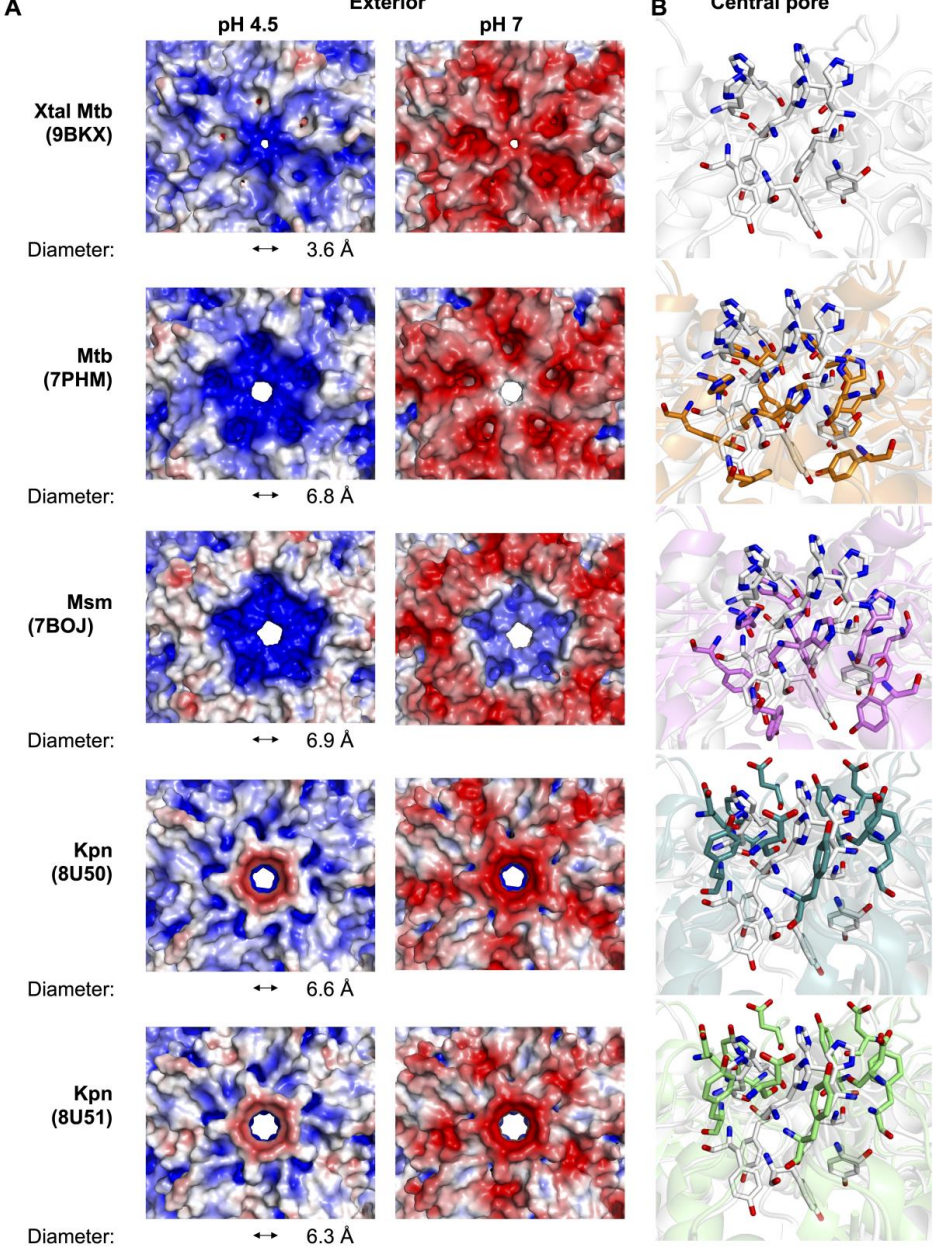
Five-fold major pore and surrounding exterior surface of Mtb-Enc shell. (**A**) The electrostatic exterior surfaces of the five-fold point of symmetry at pH 4.5 and pH 7.5, generated by pdb2qr and APBS [41,42]. Surfaces are colored according to charge, ranging from red (negatively charged) to blue (positively charged). Pore diameter determined by CAVER [43]. (**B**) Stick representation of the charged residues that line the central pore as viewed from the exterior. For comparison with cryo-EM structures, the crystal structure is shown in white.

The Enc icosahedral structure has locations at the Enc subunit interfaces with five-fold, three-fold, and two-fold symmetry (Figure 1), and at these interfaces, we observe five-fold, three-fold, and two-fold pores, respectively (Figure 1C). While investigating these pores in the Mtb-Enc•DyP structure (PDB ID 9BKX), we found that the three-fold and two-fold pores were minimally open to solvent and that the five-fold pore is only slightly open (Figure 1C). In contrast, the five-fold pores in cryo-EM structures of Mtb-Enc (PDB IDs 7PHM, 8IKA, and 8PYS) are significantly more open (Figure 2A).

We further investigated these features using MOLE (v. online) [44] and CAVER (v. 3.0 PyMOL plugin) [43], tools that allow for the characterization of channels, tunnels, and pores in macromolecular structures. Specifically, we compared the smallest diameter or bottleneck, hydropathy, and polarity of various pores in the different Enc structures. We used CAVER to determine the smallest diameter of each Enc pore [43] and found that Mtb-Enc structures solved without DyP cargo (PDB ID 7PHM and 8IKA) have bottlenecks in the pore with a diameter of 6.8 and 8.5 Å, respectively (Figure 2A). The Mtb-Enc structures solved with DyP cargo (PDB ID 9BKX and 8PYS) have bottleneck diameters of 3.6 Å and 7.3 Å, respectively, while the Msm-Enc•DyP structure (PDB ID 7BOJ) has a bottleneck 6.9 Å in diameter (Figure 2A). Interestingly, the *Haliangium ochraceum* Enc has been shown to adopt both closed and open five-fold pores in the presence of its native cargo, ferritin [11], and apo-Enc from *Acidipropionibacterium acidipropionici* Enc can also adopt both open and closed five-fold pore conformations [26]. For *H. ochraceum* Enc•ferritin, the five-fold pore expands from a closed conformation diameter of 5 Å to the open conformation diameter of 15 Å [11]*,* and for *A. acidipropionici* Enc, the closed 5 Å diameter conformation opens to 20 Å in diameter [26]. While the five-fold pore of mycobacterial Enc has been captured in a variety of conformations, we do not know if mycobacterial Enc opens to a similar extent to *H. ochraceum* Enc•ferritin or apo *A. acidipropionici* Enc. Notably, the crystal structure of Mtb-Enc•DyP described here has the smallest mycobacterial five-fold pore described to date at 3.6 A (Figure 2A).

When investigating the five-fold pore, we also found that there are only two polar residues that face into the pore: His187 and Tyr189 (Figure 2B and Figure S2). Strikingly, there is little variation in the position of these residues in the three cryo-EM Mtb-Enc structures (PDB IDs: 7PHM, 8IKA, and 8PYS). However, in our crystal structure we found a dramatic difference in the loop bearing these residues (Figure S3B). In the cryo-EM structures, His187 and Tyr189 are stacked on top of each other, symmetrically presenting His187-N_ε_2 and Tyr189-OH into the pore (Figure 2B). In the crystal structure, the C_α_ of His187 is translated ~5–6 Å upwards, resulting in the orientation of its carbonyl oxygen directly into the pore, and Tyr189 is angled ~45–50° along the pore and translated such that the hydroxyl group is ~6–7 Å away from its cryo-EM position. The result is a significant increase in polarity around the pore (25.5 vs. 3.9–18.6 as described by the Zimmerman scale, where the most polar amino acids have the highest value) for the crystal structure relative to the cryo-EM structures [44].

Recently, structures of Kpn-Enc were solved with and without non-native DyP-TP cargo (PDB IDs 8U50 and 8U51 [17]). As mentioned above, these structures represent some of the closest structural homologs of the Mtb-Enc•DyP crystal structure. When investigating the Kpn-Enc five-fold pores, we focused on the positions of their five-fold pore inward-facing polar residues, Asp187 and Tyr189 (Figure 2B and Figure S2). The sidechains of Asp187 and Tyr189 are both directed up and out of the pore, similar to His187 from the Mtb-Enc crystal structure (Figure 2B). Thus, it appears the Mtb-Enc•DyP crystal structure has captured an intermediary state in between the cryo-EM structures of Mtb- and Kpn-Enc. It is likely that the five-fold pore can sample these three conformations in both species depending on the environment.

Notably, the positions of the two polar residues appear to be independent of cargo protein internalization, as we note similar positions for DyP-bound and apo cryo-EM Enc structures from Mtb and Kpn. Interestingly, MOLE analysis [44] revealed that the Kpn-Enc five-fold pore is dramatically less polar (2.7–2.8) than the Mtb five-fold pores (3.9–25.5), likely due to the orientation of Asp187 and Tyr189, which point away from the center of the five-fold pore in the Kpn-Enc structure. The structures of Kpn-Enc also show a change in the bottleneck diameter, from 6.6 Å to 6.3 Å, with non-native DyP-TP bound (Figure 2A) [43].

To date, the native substrate(s) for DyP-type Enc nanocompartments has not been identified, and questions remain about how DyP substrate(s) is delivered. Previous experiments have shown that the Enc shell is permeable to specific ligands such as H_2_O_2_, 2,2′-azino-bis(3-ethylbenzothiazoline-6-sulfonic acid) (ABTS), 7,8-dihydroneopterin, and ferrous ammonium sulfate, but is impermeable to guaiacol [5]. It is intriguing that the large molecule, ABTS (515 g/mol and 9 × 4 Å), can enter the nanocompartment, but guaiacol (124 g/mol and 6 × 2 Å) cannot. Neither ABTS nor guaiacol could fit through the five-fold pore in any of the Mtb-, Msm- or Kpn-Enc structures, but could easily traverse the open five-fold pores of *H. ochraceum* or *A. acidipropionici* Enc discussed above [11,26]. One potential reason behind the inability of guaiacol to enter Enc is the presence of a greasy surface on its central phenol ring that has no polar groups. In contrast, both ABTS and 7,8-dihydroneopterin are well decorated with hydrophilic chemical groups. Thus, it is likely that the native substrate will present a more polar surface than guaiacol, but also that the pore responsible for substrate entry has not been captured in an open state in any of the Mtb-Enc structures.

### 3.3. Minor Pores Surrounding the Five-Fold Central Pore May Allow Small Molecules to Enter Enc

**Figure 3:**
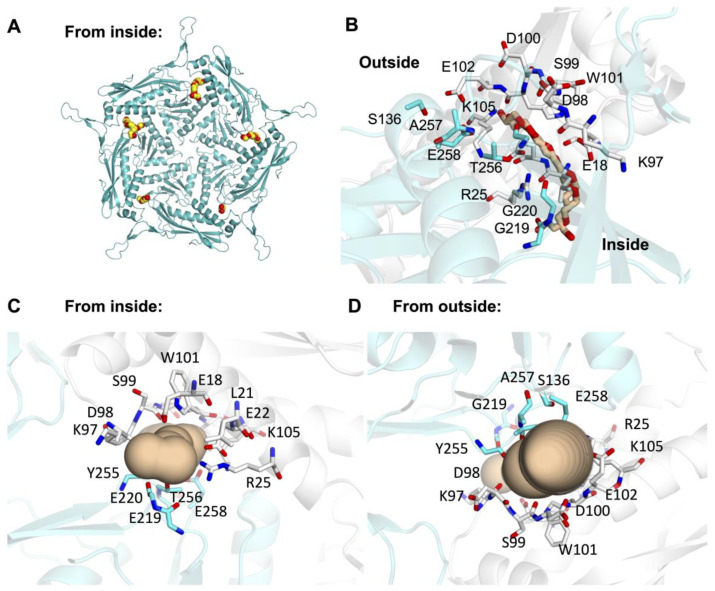
The five-fold minor pore is formed between Mtb-Enc subunits along the five-fold symmetry axis. (**A**) Mtb-Enc is shown as a cyan cartoon, and ligand occupying the minor pores are shown in sphere representation with yellow carbon and red oxygen atoms. (**B**–**D**) The minor pore at the interface of the C (cyan) and F (white) subunits. Protein is shown in cartoon and residues that line the pore are shown as sticks. The PEG molecule occupying the pore is shown as stick representation with wheat-colored carbons in (**B**), while a depiction of the pore generated in MOLE is shown in (**C**,**D**). The pore is viewed from the side in (**B**), from the interior in (**C**), and from the exterior in (**D**).

Others have previously noted the presence of small pores on the surface of Enc beyond the pores formed along symmetry axes [22]. Indeed, while investigating the surface of the crystal structure of Mtb-Enc•DyP, we found small pores at numerous places across the surface of the nanocompartment. For example, near the three-fold and two-fold pores, small pores are present along the three-fold symmetry axis or internal to individual subunits at the two-fold axis (Figure 1C). Further, when investigating areas where small molecules from the purification buffer or crystallographic condition colocalize, we noted intriguing small pores formed at the interface of neighboring subunits along the five-fold symmetry axis, which will be referred to as five-fold minor pores, while the pore formed at the five-fold symmetry axis will be referred to as the five-fold major pore. Notably, all five minor pores surrounding each five-fold major pore in the ASU are occupied by solvent molecules (Figure 3A).

The five-fold minor pores in the Mtb crystal structure are formed by a vast array of residues at the interface between two subunits. Residues that are involved in every pore are Glu18, Leu21, Glu22, Arg25, Lys97, Asp98, Ser99, Asp100, Trp101, Gly220, Tyr255, Thr256, Ala257, and Glu258 (Figure 3B–D and Figure S2). In addition to these residues, several pores also comprise Glu102, Lys105, and Ser136. The resulting pore has, on average, a bottleneck diameter of 3.4 ± 0.8 Å, with −1.8 ± 0.4 hydropathy and 26.6 ± 4.1 polarity (Table 2) [44]. DyP has peroxidase activity, converting H_2_O_2_ to water, when it degrades dye-colorizing-like substrates. H_2_O_2_ has a mean diameter of 2.5–2.8 Å and is able to traverse aquaporin through a pore with a diameter of 3 Å [45]. This suggests that these five-fold minor pores would allow for the passage of H_2_O_2_ through Enc to its cargo protein, DyP.

**Figure 4:**
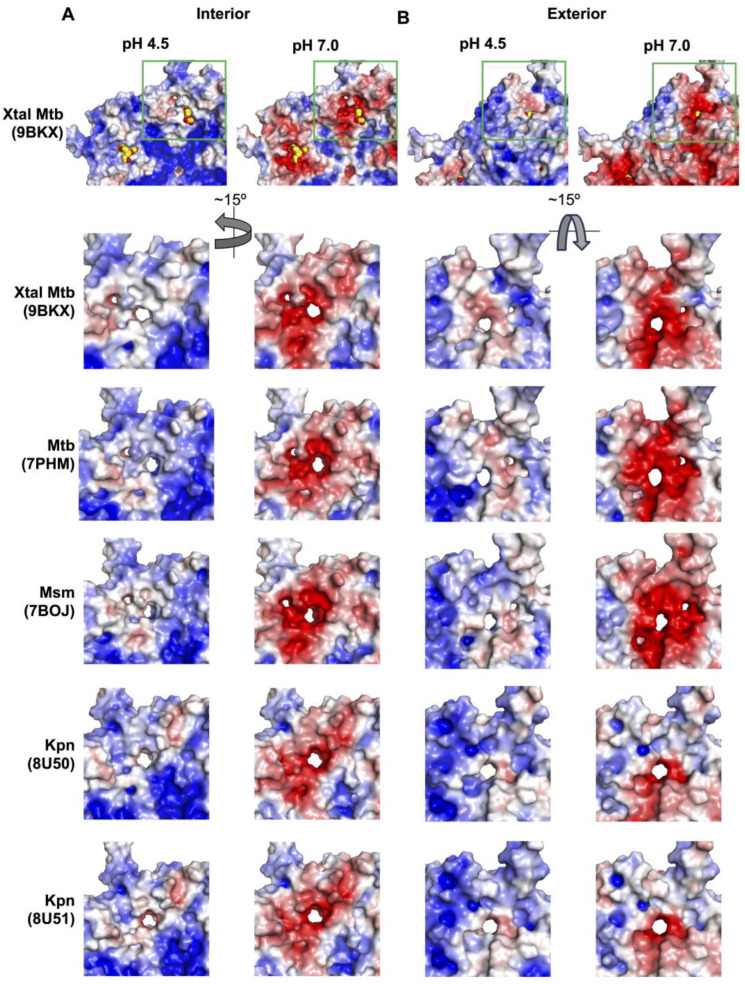
The five-fold minor pores across Enc structures. Shown here is the interior (**A**) and exterior (**B**) electrostatic surface of various Mtb, Msm, and Kpn cryo-EM structures in addition to the Mtb crystal structure at the five-fold minor pores. Notably, in the crystal structure the minor pores are occupied by small molecules (shown as spheres with yellow carbons). The green inset box indicates the region depicted below for comparison with the other structures. Surfaces are colored as in Figure 2A.

Indeed, five-fold minor pores are present in each Mtb-, Msm-, and Kpn-Enc structure (Figure 4, Table 2). In each, we see similar features in terms of diameter, hydropathy, and polarity, indicating these five-fold minor pores could also accommodate the passage of H_2_O_2_. Further, the majority of residues that line the minor five-fold pores are conserved across species; these include Glu22, Arg25, Asp98, Ser99, Asp100, Trp101, Gly220, Thr256, and Glu258 (Figure S2).

### 3.4. Biologically Relevant Electrostatic Surfaces Show Differences at the Five-Fold Major Pore Across Species

Previous discussions of the molecular surface of Enc predominately highlight surface electrostatics at neutral pH [7,8,15,16,17,18,19,20,24]. However, studies have shown that DyP is most active at low pH [48,49,50] and Mtb-Enc•DyP is part of the oxidative stress response and is also most active at low pH [5,6]. Thus, we decided to examine the electrostatic surface of Mtb-Enc at pH 4.5, which likely mimics physiological pH when Mtb encounters oxidative stress, as well as at neutral pH. Protein surfaces were modeled at pH 4.5 and 7 using PDB2PQR and the electrostatic surface was calculated in APBS [41,42].

When comparing mycobacterial Enc•DyP nanocompartments at pH 4.5 and pH 7, we observe an overall change around the five-fold major pore (Figure 2A and Figure S3A). At pH 4.5, the exterior surface of the pore is positively charged for all available mycobacterial structures. Similarly, the interior surface of the pore is positively charged for the majority of structures, excluding the crystal structure, which has a neutral to slight negatively charged ring at the interior of the pore (Figure S3A). At pH 7, however, the exterior surface of the pore varies quite widely across mycobacterial species, presenting a strongly negative charge for the crystal structure, a neutral to negative surface for the cryo-EM Mtb structure, and a positively charged surface for the Msm structure (Figure 2A). In contrast to the exterior, the interior surface of the pore in the mycobacterial structures at pH 7 is strongly negatively charged or neutral (Figure S3A). The difference in His187 and Tyr189 orientation between the crystal and cryo-EM structures described above likely explains the altered electrostatic surface charge observed for the crystallographic five-fold major pore relative to the cryo-EM pores (Figure 2 and Figure S3A).

Interestingly, when comparing the electrostatic surfaces between mycobacterial Enc structures and Kpn-Enc, we note marked differences (Figure 2A and Figure S3A). Strikingly, in Kpn-Enc (PDB ID 8U50/8U51), the exterior surface of the pore is strongly negative at pH 7 and negative to neutrally charged at pH 4.5, while the interior surface of the pore is positively charged at both pHs (Figure S3A). Again, the varied charges at the electrostatic surfaces are likely a reflection of the differences in loop orientation (Figure S3B) and polar residues (Kpn-Asp187 vs. Mtb-His187) at the five-fold pore (Figure 2A). The species variation in the presented charge at the five-fold major pore likely indicates differences in the types of molecules that can traverse the five-fold major pore and could indicate that a common substrate such as H_2_O_2_ does not utilize the five-fold major pore across bacterial species.

If we compare the electrostatic surface at the five-fold minor pores amongst a series of Enc nanocompartment structures, we find that at pH 7, the mycobacterial structures all show strong negatively charged surfaces on the exterior and interior of the shell (Figure 4). Interestingly, for Kpn-Enc structures, we see a strong negatively charged surface at the interior, but a mixture of negatively charged and neutral surfaces on the exterior of the shell. However, at pH 4.5, the five-fold minor pores are more neutral; in some cases, the surface of the five-fold minor pore displays mixed charges (e.g., PDB IDs 7PHM, 7BOJ, and 8U50) and in others it is slightly negative (e.g., PDB IDs 9BKX and 8U51). Interestingly, this difference does not follow species or sequence variation, but rather DyP loading: the mixed-charge five-fold minor pores are found in *apo* Mtb- and Kpn-Enc structures, and the slightly negative five-fold minor pores are found in DyP-loaded Mtb-, Msm-, and Kpn-Enc structures. This difference could indicate the presence of the cargo protein signaling for, or directing, the internalization of the DyP substrate (i.e., H_2_O_2_) through the five-fold pore.

### 3.5. Recognition of Mtb-DyP-TP by Mtb-Enc

**Figure 5:**
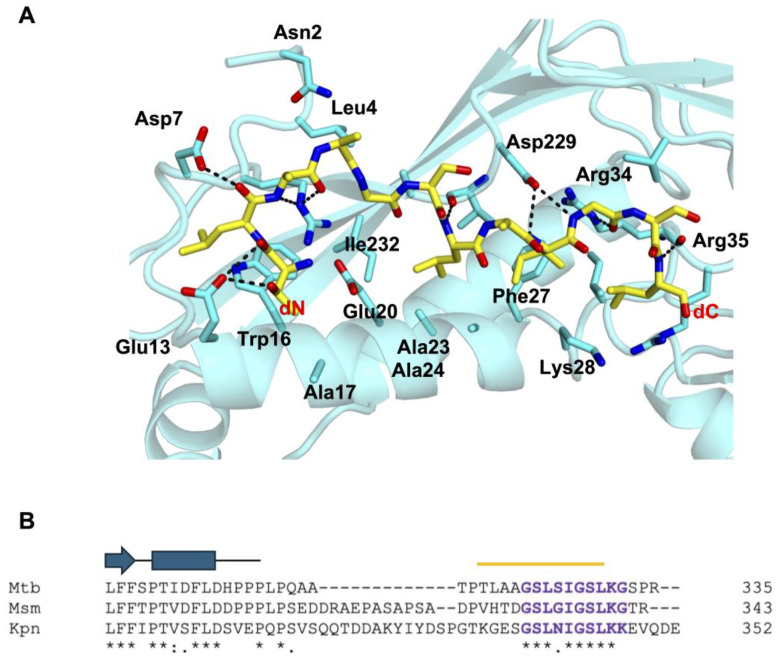
Enc recognition of the C-terminal targeting peptide (TP) of DyP. (**A**) Enc residues that coordinate the TP (subunit i, yellow stick) are shown. Enc (subunit I) is displayed as a cyan cartoon with residues involved in TP binding shown as sticks. H-bond contacts are highlighted as dashed black lines between Enc residues and DyP-TP. (**B**) A sequence alignment of the C-terminus of DyP for Mtb*,* Msm*,* and Kpn was generated by Clustal Omega [51]. A cartoon representation of the secondary structure elements for characterized DyP proteins is shown above the alignment, and a yellow line indicates the sequence modelled for DyP-TP-i (shown in (**A**)). Mtb-DyP is missing residues upstream of the DyP-TP when compared to DyP from other species. Symbols below the alignment indicate the degree of sequence conservation, where * denotes fully conserved residues, : strongly similar residues, and . weakly similar residues.

As mentioned above, nine Mtb-DyP-TPs were modeled in the ASU of the Mtb-Enc•DyP structure (Figure S1). On average, Mtb-Enc stabilizes bound Mtb-DyP-TP through 2.6 H-bonds. These H-bonds typically involve the carbonyl of Mtb-Enc-Arg34 with the backbone nitrogen of Mtb-DyP-TP-Leu330 (78% occurrence), the sidechain NH_2_ nitrogen of Mtb-Enc-Arg34 with the carbonyl of Mtb-DyP-TP-Ile327 or Mtb-DyP-TP-Leu330 (78%), and the carbonyl of Mtb-Enc-Val230 with the backbone nitrogen of Mtb-DyP-TP-Leu325 or Mtb-DyP-TP-Ser326 (44%) (Figure 5A and Figure S2).

H-bond coordination of Mtb-DyP-TP involves a peptide sidechain in only one occurrence, where the terminal nitrogen of Lys331 in peptide s is coordinated by a carboxyl oxygen of Mtb-Enc-Asp202. Notably, peptide s is the only peptide where electron density of the Lys331 sidechain is observed. Thus, apart from Mtb-DyP-TP-Lys331, it seems unlikely there is much sequence-specific recognition of the DyP-TP by Mtb-Enc through polar interactions.

Mtb-Enc does, however, coordinate the Mtb-DyP-TP sequence through sidechain interactions. The terminal nitrogen atoms (NH_1_/NH_2_) of Mtb-Enc-Arg34 are involved in coordinating five of the nine peptides, while the N_ε_ atom coordinates the carbonyl of Mtb-DyP-TP-Gly328 in another two peptides (Figure 5A). A carboxyl oxygen atom of Mtb-Enc-Asp229 coordinates the backbone nitrogen of Mtb-DyP-TP-Gly328 and, as mentioned above, a carboxyl oxygen atom of Mtb-Enc-Asp202 coordinates Mtb-DyP-TP-Lys331-N**_ζ_**.

While there are few H-bonds formed between Mtb-DyP-TP and Mtb-Enc, there is a vast network of nonbonded contacts, including hydrophobic and van der Waals interactions, ranging from 33 nonbonded contacts for peptide r to 87 for peptide i (the longest peptide modeled). Consistent nonbonded contacts are provided by Mtb-Enc Ala24, Phe27, Lys28, Ile31, Arg34, Arg35, Val39, Asp229, and Val230 (Figure 5A and Figure S2).

Overall, the DyP-TPs bound in the Kpn- and Mtb-Enc structures align well. Specifically, Mtb-DyP-TP-g, i, p, r, s, and t all align quite closely to the Kpn-DyP-TPs (Figure S1D,E). As mentioned above, Mtb-DyP-TP-i extends past most of the peptides at its N-terminal end. A few Mtb-DyP-TPs diverge from the others in their conformation: peptides d, h and q. Mtb-DyP-d diverges at the peptide’s C-terminus, while h and q are quite similar but diverge at their N-terminal end (Figure S1B,D). In summary, the majority of the Mtb-DyP-TPs are observed in a similar binding state to Kpn-DyP-TP; however, Mtb-DyP-TP-d, -h, and -q show alternative binding states.

The DyP-TP sequence is almost entirely conserved between Mtb-, Msm-, and Kpn-DyP, except for Mtb-DyP Ser326, which is a glycine (Gly335) in Msm, and an asparagine (Asn341) residue in Kpn (Figure 5B). A mutation analysis was carried out to test the residues required for recognition of the Kpn-DyP-TP (S^337^GSLNIGSLKK^347^) [17]. The results showed that the Kpn-DyP-TP bulky, hydrophobic residues (Leu340, Ile342, and Leu345), the C-terminal lysine residue (Lys346), the internal glycine residue (Gly343), and the serine residues (Ser339/Ser344 double mutant), when mutated to alanine, completely ablated binding of Kpn-DyP-TP to Kpn-Enc [17]. Notably, all these residues are conserved across Mtb-, Msm-, and Kpn-DyP (Figure 5); thus, we predict that these residues are important for DyP cargo loading in mycobacterial species as well.

Interestingly, Kpn-DyP-TP Asn341—the only nonconserved residue—is not an essential determinant of Kpn-DyP/Enc recognition, as Asn341Ala-DyP displayed only a 38% reduction in Kpn-Enc binding [17]. Mutations of Kpn-Enc residues within the Kpn-Dyp-TP recognition pocket were also evaluated [17]. It was observed that Kpn-Enc-Arg34 was essential, while Asp229 and Ile230 were important for Kpn-DyP-TP recognition. Notably, these Enc residues are conserved across species, although Kpn-Enc-Ile230 is a valine (Val230) residue in mycobacterial species, and are implicated in DyP-TP recognition in the crystal structure of Mtb-Enc•DyP (Figure S2). Thus, we would expect similar interaction impairments with the mutation of these three Enc residues in mycobacterial species.

Downstream of DyP-TP, there is no homology in the number or sequence of the final few residues (Figure 5B), suggesting that they are not important in Enc recognition. When comparing the amino acid sequence upstream of DyP-TP, there is a substantial gap in the alignment before Mtb-DyP-Thr317 (Kpn-Gly332). Here, Kpn-DyP has a string of charged and aromatic residues and Msm-DyP has a proline/alanine/serine-rich region which are absent from Mtb-DyP. Prior to this break in sequence similarity, the DyP proteins have high sequence conservation (Figure 5B). Notably, published structures of Enc-associated DyP do not extend to the C-terminal TP sequence nor do they contain the extra sequence present in Kpn- and Msm-DyP that is absent in Mtb-DyP: the Kpn-DyP structure ends at Pro315 (PDB ID 8U4Z; [17]) and Msm-DyP ends at Pro311 (PDB ID 7BOK; [18]). Thus, we do not know if sequence differences beyond the DyP compact domain will result in any differences in secondary structure. However, it is most likely that this region of DyP (downstream of Kpn-Pro315 and Msm-Pro311) is an unstructured loop. These differences in length and charge in these disparate stretches of the loop region between the DyP compact domain and the DyP-TP recognition sequence could be important for DyP/Enc interactions across species.

Interestingly, cryo-EM studies of Msm- and *B. linens* Enc with DyP cargo show localization of the DyP cargo to the Enc three-fold axis [22,52]. The authors of these papers speculate that the three-fold pore could be responsible for delivering substrate directly to DyP. It is interesting to speculate if such colocalization occurs in Mtb; however, it does not appear to as we do not see the colocalization of TP binding restricted to the three-fold Enc axis. Indeed, as the linker between the DyP compact domain and its TP is significantly shorter in Mtb (Figure 5B) than in other DyP proteins, we might hypothesize that this truncated linker prevents the tri-peptide interaction between the Enc three-fold axis and DyP-TP described for Msm and *B. linens* Enc [22,52].

## 4. Conclusions

Several structures of DyP-type Enc nanocompartments have been investigated to date, but many questions remain unanswered. The crystal structure of Mtb-Enc•DyP discussed herein represents the first mycobacterial structure of a native DyP-loaded compartment where DyP residues are resolved. Exactly how DyP is recognized by the nanocompartment will require further exploration. Intriguingly, the native substrate of DyP remains a mystery. Additionally, how this unknown substrate enters the Enc shell—using the five-fold major or three-fold pore, through the five-fold minor pores highlighted here, or using one of the uncharacterized pores in the surface of the shell—and how it is delivered to the DyP cargo also warrant further investigation. The crystal structure of Mtb-Enc•DyP presented here provides useful direction for these future research efforts. As Enc proteins represent attractive biotechnology systems for drug design and drug delivery [29,30], furthering our understanding of Enc proteins is important and ultimately may provide future avenues towards anti-mycobacterial therapeutics.

## Figures and Tables

**Table 1:** Data collection and refinement statistics for Mtb-Enc•DyP crystal structure.

**Wavelength of collection**	1 Å
**Data collection**	SSRL 7-1
Space group	P 21 3
Cell dimensions	
a, b, c (Å)	313.5, 313.5, 313.5
α, β, γ (°)	90, 90, 90
Resolution (Å)	54.56–3.15 (3.20–3.15) ^1^
R_merge_^2^	0.284 (0.887)
I/σI	7.4 (1.4)
Completeness (%)	99.4 (99.8)
Redundancy	4.5 (4.5)
**Refinement**	
Resolution (Å)	49.0–3.15 (3.26–3.15)
Total reflections	790,230 (38,895)
Unique reflections	174,711 (8661)
R_work_/R_free_^3^	20.2/23.3 (28.2/32.8)
Ramachandran favored (%)	96.8
Ramachandran outliers (%)	0.2
Enc subunits in ASU	20
DyP peptides in ASU	9
No. atoms	42,837
Protein	41,266
Ligands	1179
Water	392
B-factors (Å^2^)	
Protein	41.9
Ligands	61.3
Water	26.3
Root mean square deviations	
Bond lengths (Å)	0.003
Bond angles (°)	0.6
PDB ID	9BKX

^1^ Values within parentheses refer to the highest resolution shell; ^2^ R_merge_ = ∑∑|I_hkl_ − I_hkl_(j)|/∑I_hkl_, where I_hkl_(j) is observed intensity and I_hkl_ is the final average value of intensity. ^3^ R_work_ = ∑||F_obs_| − |F_calc_||/∑|F_obs_| and R_free_ = ∑||F_obs_| − |F_calc_||/∑|F_obs_|, where all reflections belong to a test set of 5% data randomly selected in Phenix 1.21-5207.

**Table 2:** Characteristic measurements for the five-fold minor pore—bottleneck diameter, hydropathy, and polarity—as calculated by MOLE for several Enc structures [44].

Structure	PDB ID	Bottleneck Diameter (Å)	Hydropathy [46]	Polarity [47]
Mtb-Enc with DyP	9BKX	3.4 ± 0.8	−1.8 ± 0.4	26.6 ± 4.1
Mtb-Enc with DyP	8PYS	2.8 ± 0.3	−2.3 ± 0.3	30.5 ± 2.9
Mtb-Enc	7PHM	3.4 ± 0.6	−2.2 ± 0.3	29.2 ± 3.9
Mtb-Enc	8IKA	3.5 ± 0.7	−0.6 ± 1.0	15.7 ± 11.8
Msm-Enc with DyP	7BOJ	2.2 ± 0	−0.7 ± 0.0	14.5 ± 0.0
Kpn-Enc	8U50	4.0 ± 0.8	−2.5 ± 0.9	20.0 ± 5.7
Kpn-Enc with SUMO-DyP-TP	8U51	4.0 ± 1.4	−2.3 ± 1.2	14.8 ± 1.5

## Data Availability

The data that support the findings of this study are openly available at the Protein Data Bank, PDB ID 9BKX.

## Supplementary Materials

The following supporting information can be downloaded at: https://www.mdpi.com/article/10.3390/microorganisms12122465/s1, Table S1: Structural homologs for Mtb-Enc subunit from Mtb-Enc•DyP using Dali; Figure S1: The crystallographic asymmetric unit (ASU) for the Mtb-Enc•DyP complex and comparison of bound Mtb-DyP-TP conformations; Figure S2: Sequence alignment for Enc proteins; Figure S3: Five-fold major pore of the Enc shell.
