# Supplementary material for: Structural Characterization of Mycobacterium tuberculosis Encapsulin in Complex with Dye-Decolorizing Peroxide
Source: Microorganisms. 2024 Nov 30;12(12):2465. doi: 10.3390/microorganisms12122465 (PMC11676171; doi:10.3390/microorganisms12122465)
Supplement: Supplementary file 1 [file microorganisms-12-02465-s001.zip › microorganisms-3340063-supplementary.pdf]

Table S1. Structural homologs for Mtb-Enc subunit from Mtb-Enc•DyP using Dali [1].

**Structural homolog**	**Sym**[1]	**Identity (%)**	**PDB ID code**	**Z-score**	**rmsd**[2]**, Å**	**Reference**
Mycobacterium hassiacum Enc	T1	81	6I9G	37.1	0.39 (264 atoms)	[2]
Mycobacterium tuberculosis (Mtb) Enc	T1	100	7PHM	36.8	0.82 (264 atoms)	N/A
Mtb Enc with DyP	T1	100	8PYS	36.8	0.83 (264 atoms)	N/A
Mtb Enc	T1	100	8IKA	36.3	0.81 (264 atoms)	N/A
Mycobacterium smegmatis Enc with DyP	T1	82	7BOJ	36.3	0.90 (264 atoms)	[3]
Mtb Enc with unknown cargo	T1	100	7P1T	36.2	0.90 (264 atoms)	N/A
Klebsiella pneumoniae (Kpn) Enc with SUMO-DyP-TP	T1	55	8U51	34.3	0.91 (264 atoms)	[4]
Brevibacterium linens Enc	T1	58	7BCV	34.3	0.98 (264 atoms)	[5]
Kpn Enc	T1	58	8U50	34.2	0.90 (264 atoms)	[4]
Haliangium ochraceum Enc pentamer with Ftn, open	T1	39	7OEU	33.1	1.27 (264 atoms)	[6]
Acidipropionibacterium acidipropionici Enc, pH 8	T4	49	8DNA	33.0	1.16 (264 atoms)	[7]
A. acidipropionici Enc, pH 7.5	T4	49	8DN9	32.8	1.12 (256 atoms)	[7]
H. ochraceum Enc pentamer with Ftn, closed, peptide modeled	T1	38	7OE2	32.2	1.42 (264 atoms)	[6]
H. ochraceum Enc	T1	38	7ODW	32.1	1.42 (264 atoms)	[6]
A. acidipropionici Enc, pH 7.5	T4	46	8DNL	30.5	1.73 (256 atoms)	[7]
Thermotoga maritima Enc	T1	35	7MU1	28.5	2.21 (256 atoms)	[8]
T. maritima Enc with Flp3	T1	35	3DKT	27.8	2.24 (256 atoms)	[9]
T. maritima Enc	T1	35	7KQ5	27.4	2.27 (256 atoms)	[10]
T. maritima Enc	T1	35	7K5W	27.1	2.35 (256 atoms)	[11]
T. maritima Enc, pore mutant	T1	35	7LIT	25.3	2.41 (248 atoms)	[12]
T. maritima Enc, pore mutant	T1	35	7LIS	25.2	2.64 (256 atoms)	[12]
Myxococcus xanthus Enc	T1	21	7S21	23.5	4.59 (256 atoms)	[13]

M. xanthus EncA with EncB	T3	23	7S2T	23.1	7.07 (240 atoms)	[13]
M. xanthus EncA with EncC	T3	23	7S4Q	22.8	7.18 (240 atoms)	[13]
M. xanthus EncA	T3	22	7S20	22.6	7.15 (240 atoms)	[13]
Quasibacillus thermotolerans Enc with IMEF4	T4	19	6NJ8	22.6	3.81 (192 atoms)	[14]
M. xanthus EncA with SNAP-tag cargo protein	T3	23	8TK7	22.2	7.15 (240 atoms)	[15]
M. xanthus EncA	T3	22	4PT2	21.6	7.05 (240 atoms)	[16]
Pyrococcus furiosus virus-like particle	T3	20	2E0Z	20.1	5.35 (224 atoms)	[17]

[1] Triangulation number for icosahedral: T = 1 (60 subunits), T = 3 (180 subunits), or T = 4 (240 subunits) [9,14,17].
[2] root mean square deviation (rmsd) by cealign in PyMOL measured over Cα atoms [18].
[3] Ferritin-like protein (Flp) targeting peptide (TP) modelled into the density. But true identity of peptide is unconfirmed.
[4] Iron-Mineralizing Encapsulin-Associated Firmicute (IMEF).

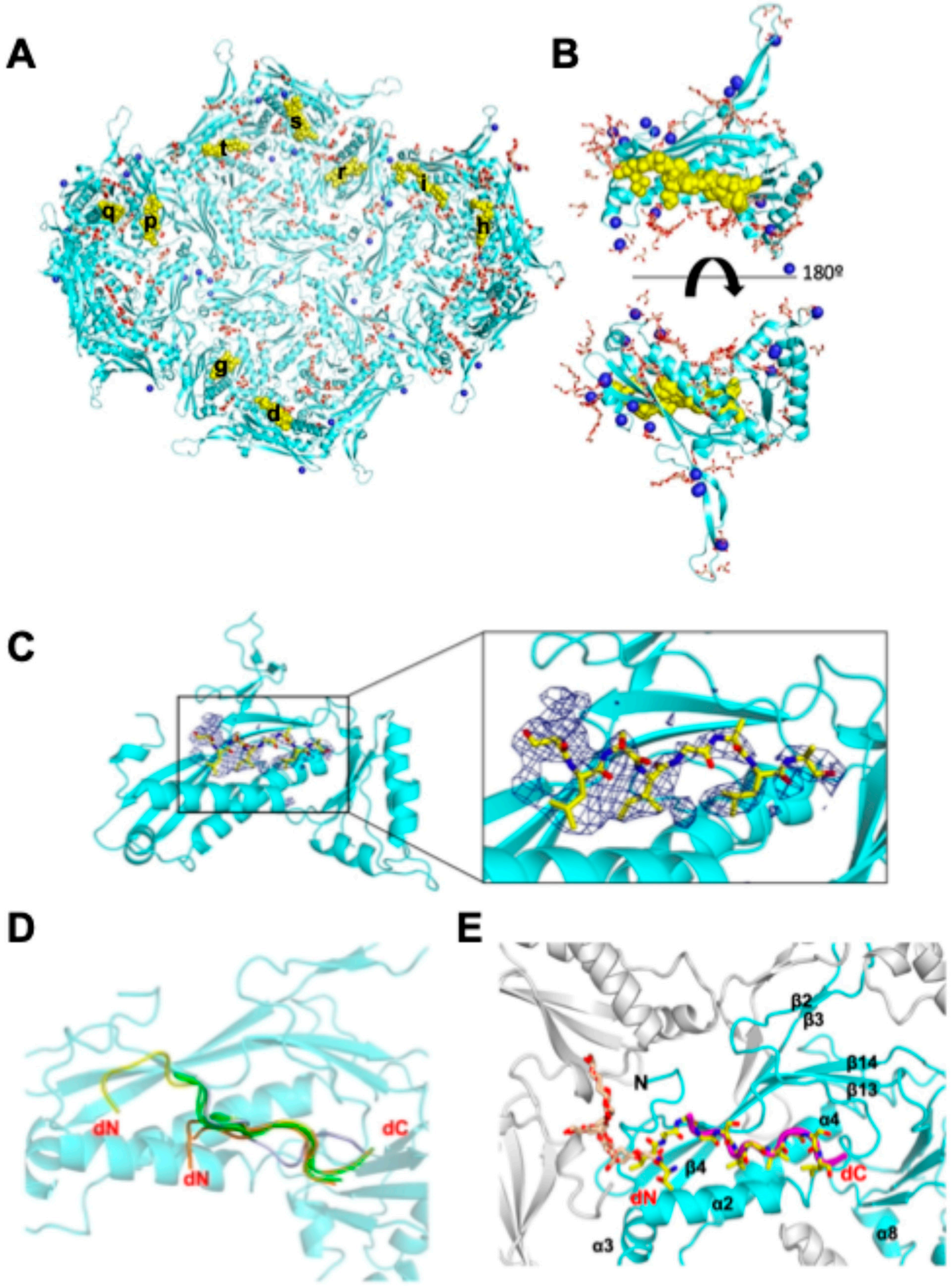

A
t
s
r
i
h
q
p
g
d
B
180º
C
D
dN
dN
dC
E
β2
β3
β14
β13
N
α4
β4
dN
dC
α2
α3
α8

Figure S1. The crystallographic asymmetric unit (ASU) for Mtb-Enc•DyP complex and comparison of bound DyP-TP conformations.

A. The ASU contains 20 subunits (cyan cartoons), 9 DyP-TPs (yellow sphere), and many bound small molecules (ligands or PEG/glycerol shown in stick with wheat carbon and red oxygen atoms) and nickel ions (blue spheres).
B. Alignment of all the subunits from the ASU shows colocalization of the peptides, but also areas with high incidence of bound small molecules (PEG, glycerol and nickel ions). Molecules are colored as in (A). Only subunit A is shown in cartoon representation.
C. Electron density (blue mesh) for peptide t was generated as a Polder map [19] and is shown at 3 σ. An inset shows a closer view of the peptide binding site. Protein and peptide are colored as in panel B.
D. Enc subunits bound to DyP-TP were aligned, and DyP-TP is shown for comparison alongside Enc chain T (cyan). A few distinct conformations are grouped by color: lavender (DyP-TP-d), green (DyP-TP-g, -p, -r, -s, and -t), orange (DyP-TP-h and -q), and yellow (DyP-TP-i).
E. Comparison of peptide binding locations for Mtb-DyP (chain I, yellow sticks) and Kpn-DyP (pink cartoon). For comparison, the structure of the Kpn-Enc•SUMO-DyP-TP complex (PDB ID 8U51) was aligned to the Mtb complex structure shown in cartoon. In several subunits of Mtb-Enc we see small molecules bound near the N-terminus of bound DyP-TP (dN). Alignment of Mtb-Enc subunits shows colocalization of small molecules near the dN of the TP-binding site, these small molecules are shown in stick representation with carbons colored wheat.

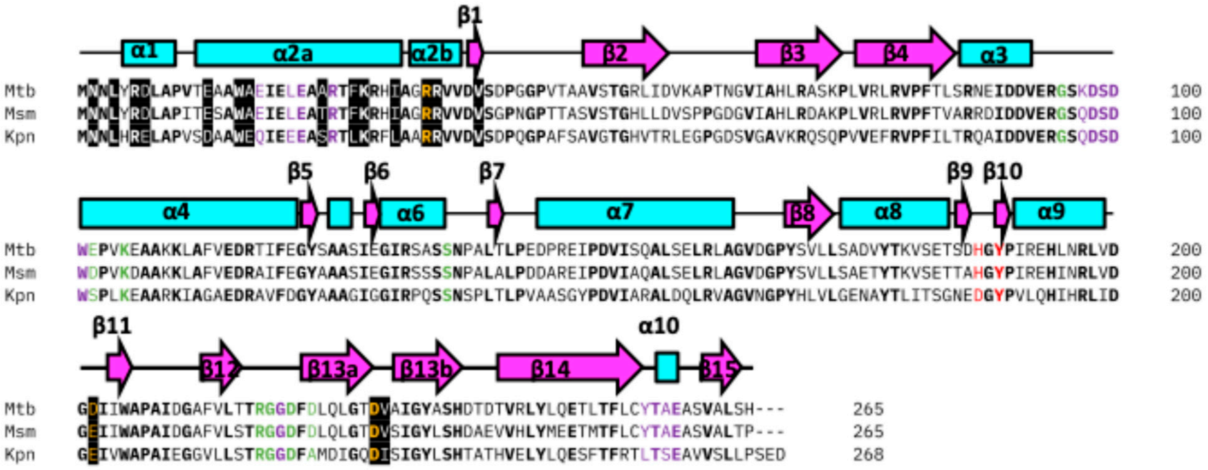

Figure S2. Sequence alignment for Enc proteins. Sequence alignment was generated in Clustal Omega [20] with Enc from *M. tuberculosis* (Mtb), *M. smegmatis* (Msm) and *K. pneumoniae* (Kpn). Above the sequence alignment is the secondary structure elements for Mtb-Enc from the crystal structure with β-strands in pink and α-helices in cyan. Residues involved in peptide coordination are boxed in black: residues involved in H-bonds are yellow and non-bonded contacts are white; the five-fold major pore charged residues are colored red; five-fold minor pore residues that are involved in every subunit are colored purple while residues involved in only some subunits are colored green. Residues conserved across species are bolded.

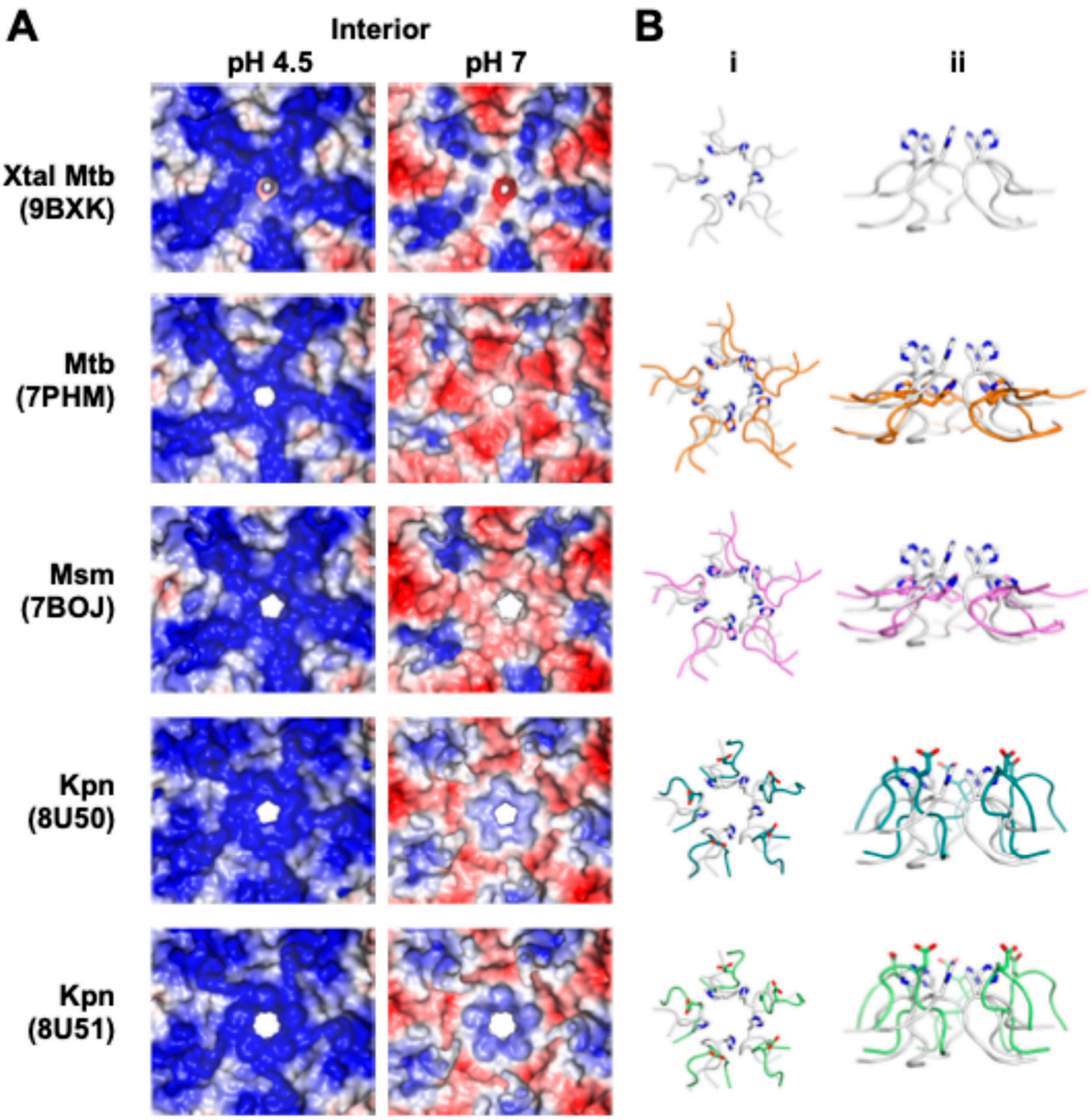

Figure S3. Five-fold major pore of the Enc shell.

A. The electrostatic interior surface of the five-fold point of symmetry at pH 4.5 and pH 7.5, generated by pdb2qr and APBS [21,22].

B. Cartoon depiction of the central loops bearing the charged residues in the five-fold major pore. The view of (i) is similar to Figure 2B and (ii) is a clock-wise 90° rotation of (i) around the x-axis. For the mycobacterial Encs, the sidechain of His187 is shown in stick; for Kpn-Enc, Asp187. For comparison with cryo-EM structures, the crystal structure is shown in white.

References

1. Holm, L.; Rosenstrom, P. Dali server: conservation mapping in 3D. *Nucleic Acids Res.* **2010**, *38*, W545-W549, doi:10.1093/nar/gkq366.
2. Lončar, N.; Rozeboom, H.J.; Franken, L.E.; Stuart, M.C.A.; Fraaije, M.W. Structure of a robust bacterial protein cage and its application as a versatile biocatalytic platform through enzyme encapsulation. *Biochemical and Biophysical Research Communications* **2020**, *529*, 548-553, doi:10.1016/j.bbrc.2020.06.059.
3. Tang, Y.; Mu, A.; Zhang, Y.; Zhou, S.; Wang, W.; Lai, Y.; Zhou, X.; Liu, F.; Yang, X.; Gong, H.; et al. Cryo-EM structure of Mycobacterium smegmatis DyP-loaded encapsulin. *Proceedings of the National Academy of Sciences of the United States of America* **2021**, *118*, 1-8, doi:10.1073/pnas.2025658118.
4. Jones, J.A.; Andreas, M.P.; Giessen, T.W. Structural basis for peroxidase encapsulation inside the encapsulin from the Gram-negative pathogen Klebsiella pneumoniae. *Nature Communications* **2024**, *15*, doi:10.1038/s41467-024-46880-x.
5. Putri, R.M.; Allende-Ballestero, C.; Luque, D.; Klem, R.; Rousou, K.A.; Liu, A.; Traulsen, C.H.H.; Rurup, W.F.; Koay, M.S.T.; Castón, J.R.; et al. Structural Characterization of Native and Modified Encapsulins as Nanoplatforms for in Vitro Catalysis and Cellular Uptake. *ACS Nano* **2017**, *11*, 12796-12804, doi:10.1021/acsnano.7b07669.
6. Ross, J.; McIver, Z.; Lambert, T.; Piergentili, C.; Bird, J.E.; Gallagher, K.J.; Cruickshank, F.L.; James, P.; Zarazúa-Arvizu, E.; Horsfall, L.E.; et al. Pore dynamics and asymmetric cargo loading in an encapsulin nanocompartment. *Science Advances* **2022**, *8*, 1-12, doi:10.1126/sciadv.abj4461.
7. Jones, J.A.; Andreas, M.P.; Giessen, T.W. Exploring the Extreme Acid Tolerance of a Dynamic Protein Nanocage. *Biomacromolecules* **2023**, *24*, 1388-1399, doi:10.1021/acs.biomac.2c01424.
8. LaFrance, B.J.; Cassidy-Amstutz, C.; Nichols, R.J.; Oltrogge, L.M.; Nogales, E.; Savage, D.F. The encapsulin from Thermotoga maritima is a flavoprotein with a symmetry matched ferritin-like cargo protein. *Scientific Reports* **2021**, *11*, 1-10, doi:10.1038/s41598-021-01932-w.
9. Sutter, M.; Boehringer, D.; Gutmann, S.; Günther, S.; Prangishvili, D.; Loessner, M.J.; Stetter, K.O.; Weber-Ban, E.; Ban, N. Structural basis of enzyme encapsulation into a bacterial nanocompartment. *Nature Structural and Molecular Biology* **2008**, *15*, 939-947, doi:10.1038/nsmb.1473.
10. Wiryaman, T.; Toor, N. Cryo-EM structure of a thermostable bacterial nanocompartment. *IUCrJ* **2021**, *8*, 342-350, doi:10.1107/S2052252521001949.
11. Xiong, X.; Sun, C.; Vago, F.S.; Klose, T.; Zhu, J.; Jiang, W. Cryo-EM structure of heterologous protein complex loaded thermotoga maritima encapsulin capsid. *Biomolecules* **2020**, *10*, 1-13, doi:10.3390/biom10091342.
12. Adamson, L.S.R.; Tasneem, N.; Andreas, M.P.; Close, W.; Jenner, E.N.; Szyszka, T.N.; Young, R.; Cheah, L.C.; Norman, A.; MacDermott-Opeskin, H.I.; et al. Pore structure controls stability and molecular flux in engineered protein cages. *Science Advances* **2022**, *8*, 1-12, doi:10.1126/sciadv.abl7346.
13. Eren, E.; Wang, B.; Winkler, D.C.; Watts, N.R.; Steven, A.C.; Wingfield, P.T. Structural characterization of the Myxococcus xanthus encapsulin and ferritin-like cargo system gives

insight into its iron storage mechanism. *Structure* **2022**, *30*, 551-563.e554, doi:10.1016/j.str.2022.01.008.
14. Giessen, T.W.; Orlando, B.J.; Verdegaal, A.A.; Chambers, M.G.; Gardener, J.; Bell, D.C.; Birrane, G.; Liao, M.; Silver, P.A. Large protein organelles form a new iron sequestration system with high storage capacity. *eLife* **2019**, *8*, 1-23, doi:10.7554/eLife.46070.
15. Kwon, S.; Andreas, M.P.; Giessen, T.W. Structure and heterogeneity of a highly cargo-loaded encapsulin shell. *Journal of Structural Biology* **2023**, *215*, 108022-108022, doi:10.1016/j.jsb.2023.108022.
16. McHugh, C.A.; Fontana, J.; Nemecek, D.; Cheng, N.; Aksyuk, A.A.; Heymann, J.B.; Winkler, D.C.; Lam, A.S.; Wall, J.S.; Steven, A.C.; et al. A virus capsid-like nanocompartment that stores iron and protects bacteria from oxidative stress. *The EMBO Journal* **2014**, *33*, 1896-1911, doi:10.15252/embj.201488566.
17. Akita, F.; Chong, K.T.; Tanaka, H.; Yamashita, E.; Miyazaki, N.; Nakaishi, Y.; Suzuki, M.; Namba, K.; Ono, Y.; Tsukihara, T.; et al. The Crystal Structure of a Virus-like Particle from the Hyperthermophilic Archaeon Pyrococcus furiosus Provides Insight into the Evolution of Viruses. *Journal of Molecular Biology* **2007**, *368*, 1469-1483, doi:10.1016/j.jmb.2007.02.075.
18. The PyMOL Molecular Graphics System Version 2.5; Schrödinger, LLC.
19. Liebschner, D.; Afonine, P.V.; Moriarty, N.W.; Poon, B.K.; Sobolev, O.V.; Terwilliger, T.C.; Adams, P.D. Polder maps: Improving OMIT maps by excluding bulk solvent. *Acta Crystallographica Section D: Structural Biology* **2017**, *73*, 148-157, doi:10.1107/S2059798316018210.
20. Madeira, F.; Madhusoodanan, N.; Lee, J.; Eusebi, A.; Niewielska, A.; Tivey, A.R.N.; Lopez, R.; Butcher, S. The EMBL-EBI Job Dispatcher sequence analysis tools framework in 2024. *Nucleic Acids Research* **2024**, *52*, W521-W525, doi:10.1093/nar/gkae241.
21. Jurrus, E.; Engel, D.; Star, K.; Monson, K.; Brandi, J.; Felberg, L.E.; Brookes, D.H.; Wilson, L.; Chen, J.; Liles, K.; et al. Improvements to the APBS biomolecular solvation software suite. *Protein Science* **2018**, *27*, 112-128, doi:10.1002/pro.3280.
22. Unni, S.; Huang, Y.; Hanson, R.M.; Tobias, M.; Krishnan, S.; Li, W.W.; Nielsen, J.E.; Baker, N.A. Web Servers and Services for Electrostatics Calculations with APBS and PDB2PQR SAMIR. *Journal of computational chemistry* **2011**, *32*, 1488-1491, doi:10.1002/jcc.21720.
